# Parallel Markov chain Monte Carlo - bridging the gap to high-performance Bayesian computation in animal breeding and genetics

**DOI:** 10.1186/1297-9686-44-29

**Published:** 2012-09-25

**Authors:** Xiao-Lin Wu, Chuanyu Sun, Timothy M Beissinger, Guilherme JM Rosa, Kent A Weigel, Natalia de Leon Gatti, Daniel Gianola

**Affiliations:** 1 Department of Dairy Science, University of Wisconsin, Madison, WI, USA; 2 Department of Animal Sciences, University of Wisconsin, Madison, WI, USA; 3 Department of Agronomy, University of Wisconsin, Madison, WI, USA; 4 Department of Biostatistics and Medical Informatics, University of Wisconsin, Madison, WI, USA

## Abstract

**Background:**

Most Bayesian models for the analysis of complex traits are not analytically tractable and inferences are based on computationally intensive techniques. This is true of Bayesian models for genome-enabled selection, which uses whole-genome molecular data to predict the genetic merit of candidate animals for breeding purposes. In this regard, parallel computing can overcome the bottlenecks that can arise from series computing. Hence, a major goal of the present study is to bridge the gap to high-performance Bayesian computation in the context of animal breeding and genetics.

**Results:**

Parallel Monte Carlo Markov chain algorithms and strategies are described in the context of animal breeding and genetics. Parallel Monte Carlo algorithms are introduced as a starting point including their applications to computing single-parameter and certain multiple-parameter models. Then, two basic approaches for parallel Markov chain Monte Carlo are described: one aims at parallelization within a single chain; the other is based on running multiple chains, yet some variants are discussed as well. Features and strategies of the parallel Markov chain Monte Carlo are illustrated using real data, including a large beef cattle dataset with 50K SNP genotypes.

**Conclusions:**

Parallel Markov chain Monte Carlo algorithms are useful for computing complex Bayesian models, which does not only lead to a dramatic speedup in computing but can also be used to optimize model parameters in complex Bayesian models. Hence, we anticipate that use of parallel Markov chain Monte Carlo will have a profound impact on revolutionizing the computational tools for genomic selection programs.

## Background

In recent decades, Bayesian inference has been increasingly used for analysis of complex statistical models, in part because of increased availability and performance of personal computers and workstations. However, such models are generally not analytically tractable and, hence, computationally demanding numerical techniques are inevitably required. This is especially true of Bayesian computation for genome-enabled prediction and selection, which aims at using whole-genome molecular data to predict the genetic merit of candidate animals for breeding purposes [1]. Typically, implementation of a high-dimensional model based on Markov chain Monte Carlo (MCMC) techniques is notoriously intensive in computing and often requires days, weeks, or even months of CPU (Central Processing Unit) time on personal computers and workstations [2]. Therefore, in order to overcome such computational burden, parallel computing becomes appealing [3,4].

Parallel computing operates on the principle that a large problem can be split into smaller components and solved concurrently (i.e., ”in parallel”), each on a separate processor (or CPU core) [5]. An instance of a computer program and its activities that are taking place on each processor is referred to a process. Thus, parallel computing involves activating multiple processes that concurrently carry out related computing jobs and combining results by the main “controlling” process. Parallel computing can be achieved by programming with C, C++, or Fortran, e.g., using the MPI (Message Passing Interface) library to handle inter-process communication [6]. High-performance computing communities have developed parallel programs for decades but were previously limited to programs running on expensive super-computers. In the past twenty years, interest in parallel computing has grown markedly due to physical constraints that prevent frequency scaling [5] and to the need to handle datasets of unprecedented dimensionalities that are being generated [7]. Parallel computing has now become a dominant paradigm in current computer architectures, mainly in the form of multi-core processors [8].

Parallel MCMC methods have recently been adopted in statistics and informatics [4,9] and in image processing [10] but they have not received much attention in animal breeding and genetics. There are several reasons for this gap. First, MCMC algorithms are seemingly serial, and parallelism is not as straightforward as one would expect. Second, many intensive computational tasks in breeding and genetics applications have been handled via some simple data parallelism, implemented through the “multiple-tasking” mechanism provided by multi-core Linux workstations. Multiple-tasking allows each processor to switch between tasks being executed on it, without having to wait for each task to finish, but this type of “parallel” computing is not scalable with the number of jobs. Recently, parallel MCMC algorithms and strategies have become a focal point for scientific computing in the post-genome era [4]. This is largely due to the need to handle genomic datasets of unprecedented sizes, such as genome-wide dense markers or sequences for genome-enabled selection programs [2]. With a set of whole-genome markers (say 50K SNP markers or higher density) in a model, the computing task is highly challenging, particularly with sophisticated Bayesian models via MCMC implementation [1,11-13].

In this paper, we present a technical description of parallel MCMC methods in the context of animal breeding and genetics. These algorithms typically fall into two categories: running multiple MCMC chains or parallelization within a single chain; some variants of these algorithms are discussed as well. A major purpose of this paper is to advocate the use of parallel MCMC methods, hence infusing high-performance computing technologies into animal selection programs in the post-genome era.

## Methods

### Parallel Monte Carlo methods

We start with parallel Monte Carlo methods, as a prelude to parallel MCMC. In practice, many statistical problems involve integrating over hundreds or even thousands of dimensions but usually these problems are not analytically tractable. Instead, Monte Carlo simulation methods [14] can be used to tackle high-dimensional integrals. Standard Monte Carlo integration algorithms distribute the evaluation points uniformly over the integration regions.

#### Parallel computing for evaluating integrals

To begin, consider the following integral

(1) $$E\left(p\left(\theta \right)\right)=\int p\left(\theta \right)f\left(\theta \right)d\theta \text{,}$$

for some high-dimensional *θ* with density $f\left(\theta \right)$. Suppose the integral cannot be evaluated analytically. If n realizations of *θ* can be sampled independently from $f\left(\theta \right)$ then, according to the strong law of large numbers, the sample average $\frac{1}{n}\overset{n}{\underset{i=1}{\Sigma }}p\left(\theta^{\left(i\right)}\right)$ provides an approximation to $E\left(p\left(\theta \right)\right)$ when n→∞.

Simple Monte Carlo algorithms proceed by averaging large numbers of values that are generated independently of each other. Obviously, Monte Carlo simulation is parallel in computing because it can be conducted concurrently. By parallel computing, the entire set of samples can be divided among the available CPU cores and then each core generates a portion and summarizes its local samples. After all processors have finished their tasks, a master program summarizes all the partial data and outputs the final result.

Suppose that there are *K* CPU cores that generate a total of *T* samples, each handling an equal portion of these samples. For simplicity, we assume that *T* is divisible by *K*, such that the quotient (*m = T/K*) is an integer. Then, parallel Monte Carlo simulation proceeds as follows [3]:

➔ Process 0 (master process):

(a) computes and passes *m* to each process.

➔ Each (slave) process (say *j*):

(a) simulates *m* independent realizations of θ;

(b) computes $S_{j}=\overset{m}{\underset{i=1}{\Sigma }}p\left(\theta^{\left(i\right)}\right)$_,_ and passes *S*_*j*_ back to the master program.

➔ Process 0 (master program):

(a) sums *S*_*j*_ and generates the final sum $S=\overset{K}{\underset{j=1}{\Sigma }}S_{j}\text{;}$

(b) computes the Monte Carlo estimate as $E\left(p\left(\theta \right)\right)=\frac{S}{T}\text{.}$

Note that, in this example, the master process does not involve computing the sum of a portion of the data but it actually can. Also, note that each process is given the same number of samples. This works well if all CPU cores process the data at the same speed or approximately so. In practice, however, clock frequencies (i.e., computing speed) can vary markedly among processors. Hence, it can be more effective for each processor (or CPU core) to process a different number of samples, roughly proportional to its computing speed, and then let the master program compute the weighted average of all samples obtained from the *K* cores.

#### Parallel computing of single-parameter models

A single-parameter model can serve as a building block for Bayesian modeling [15]. Consider a normal distribution with known mean *μ* and unknown variance $\sigma^{2}$ to be inferred. The data density for a vector *y* of *n* identically independently distributed (*iid*) observations is:

(2) $$p(\text{y}|\sigma^{2})\propto {\left(\sigma^{2}\right)}^{-\frac{n}{2}}\exp\left(-\frac{n}{2\sigma^{2}}S^{2}\right)\text{,}$$

where $S^{2}=\frac{1}{n}\overset{n}{\underset{i=1}{\Sigma }}{\left(y_{i}-\mu \right)}^{2}$ is the sufficient statistic. Assuming an inverse-$\chi^{2}$ prior distribution with scale$\sigma_{0}^{2}$ and $\upsilon_{0}$ degrees of freedom,

(3) $$p\left(\sigma^{2}\right)\propto {\left(\frac{1}{\sigma^{2}}\right)}^{\frac{\upsilon_{0}}{2}+1}\exp\left(-\frac{\upsilon_{0}\sigma_{0}^{2}}{2\sigma^{2}}\right)\text{,}$$

it can be shown that the posterior density of $\sigma^{2}$ is a scaled inverse-$\chi^{2}$distribution with scale $\frac{\upsilon_{0}\sigma_{0}^{2}+nS^{2}}{\upsilon_{0}+n}$ and $\upsilon_{0}+n$ degrees of freedom [15]:

(4) $$\sigma^{2}|\text{y}\sim \chi^{-2}\left(\upsilon_{0}+n,\frac{\upsilon_{0}\sigma_{0}^{2}+nS^{2}}{\upsilon_{0}+n}\right)\text{.}$$

Hence, the posterior mean of $\sigma^{2}$ is $\frac{\upsilon_{0}\sigma_{0}^{2}+nS^{2}}{\upsilon_{0}+n-2}$ for $\upsilon_{0}+n>2.$ Numerically, the posterior distribution of $\sigma^{2}$ can be inferred based on posterior samples generated from (4). Computing for this single-parameter normal model can follow exactly the same algorithm as parallel Monte Carlo simulation. Briefly, *K* parallel processes are executed, each generating a portion of the posterior samples of $\sigma^{2}\text{.}$ Then, the master process generates the final sum and computes the estimated posterior mean of $\sigma^{2}$ as a weighted average of all sample averages.

To show why the algorithm of parallel Markov chain simulation applies to parallel computing of a single-parameter model, consider equation (1). For this single-parameter normal model, for example, the marginal posterior expectation of $\sigma^{2}$ can be expressed as:

(5) $$E\left(\sigma^{2}|y\right)=\int \sigma^{2}f\left(\sigma^{2}|y\right)d\sigma^{2}\text{.}$$

Clearly, (5) implies a similar Monte Carlo implementation: if *n* samples of $\sigma^{2}$ are generated from its marginal posterior density$f\left(\sigma^{2}|y\right)\text{,}$ then, as n→∞,$E\left(\sigma^{2}|\text{y}\right)$ can be approximated by the sample average:

(6) $$\bar{\sigma^{2}}=\overset{n}{\underset{i=1}{\Sigma }}{\left(\sigma^{2}\right)}^{\left(t\right)}\to E\left(\sigma^{2}|\text{y}\right)\text{.}$$

#### Parallel computing of multiple-parameter models

Many models involve more than one unknown. Although many parameters are involved, conclusions are often drawn about one or only a few parameters at a time. In Bayesian analysis, the aim is to obtain the marginal posterior distribution of each parameter of interest. Often, we can construct the joint posterior distribution of all unknowns and then integrate this distribution over the unknowns that are not of immediate interest, leading to the desired marginal distribution of the parameter of interest.

Now, consider the normal distribution (2) but with both mean and variance unknown. Assuming prior independence of location and scale parameters, a vague prior density for *μ* and $\sigma^{2}$ is uniform on $\left(\mu ,\;\log\sigma \right)$ that is,

(7) $$p\left(\mu ,\sigma^{2}\right)\propto {\left(\sigma^{2}\right)}^{-1}\text{.}$$

Then, it can be shown that the marginal posterior distribution of $\sigma^{2}$ is a scaled inverse-$\chi^{2}$ density with n−1 degrees of freedom and scaling parameter $s^{2}$:

(8) $$p\left(\sigma^{2}|y\right)=\int N\left(\text{y}|\mu ,\sigma^{2}\right)\sigma^{-2}d\mu =\chi^{-2}\left(n-1,s^{2}\right)\text{,}$$

where $s^{2}=\frac{1}{n-1}\overset{n}{\underset{i=1}{\Sigma }}{\left(y_{i}-\bar{y}\right)}^{2}$ and $\bar{y}=\frac{1}{n}\overset{n}{\underset{i=1}{\Sigma }}{\text{y}}_{i}\text{.}$ The marginal posterior distribution of *μ* can be obtained by integrating the joint posterior density over ${\text{σ}}^{2}$_,_ leading to a student-t density:

(9) $$p\left(\mu |y\right)=\int p\left(\mu ,\sigma^{2}|y\right)d\sigma^{2}=t_{n-1}\left(\bar{\text{y}},\frac{s^{2}}{n}\right)\text{.}$$

Therefore, posterior samples for $\sigma^{2}$ and *μ* can be generated independently from the following marginal posterior distributions, for t=1,…,T iterations:

➔ Sampling $\sigma^{2\left(t\right)}$ from (8),

➔ Sampling $\mu^{\left(t\right)}$ from (9).

### Parallel Markov Chain Monte Carlo

Analytical solutions are not always available for most multiple-parameter models. Instead, MCMC simulation can be used to draw samples from the joint posterior distribution and then evaluate sampled values for the parameter(s) of interest while ignoring the values of other unknowns. MCMC methods are a variant of Monte Carlo schemes in which a Markov chain $\left\{X_{j},j=1,2\right\}$ is constructed with equilibrium distribution *π* equal to some distribution of interest, such as a posterior distribution in a Bayesian analysis [16]. Typically, the initial value is not a draw from the distribution *π* but if the chain is constructed properly, then $X_{t}\overset{d}{\to }\pi$ (here, *d* means convergence in distribution) and, under certain conditions, an estimator $\hat{h}$ converges to $h_{\pi }$ as t→∞. However, a Markov chain is sequential by nature because the distribution of $X_{t+1}$ depends on the value of $X_{t}\text{,}$ where *t* indexes the order of MCMC iterations. This introduces a difficulty to parallelization of a Markov chain.

#### Parallel MCMC by running multiple chains

A naive yet natural approach to parallel MCMC is simply to generate several independent Markov chains on different processors and then combine results appropriately [17,18]. Given that running multiple chains is simple and that they scale well with the number of available processors (or CPU cores), this type of “multiple-chain” parallelism is usually the strategy to strive for in the first instance.

Assume that we want to estimate some target distribution$p\left(X\right)$ but samples of *X* cannot be drawn directly from $p\left(X\right)\text{.}$ Instead, a Markov chain $X_{0},X_{1},\ldots$ can be generated, which, through some transition density $u\left(X_{t+1}|X_{t}\right)\text{,}$ converges to $p\left(X\right)$ at equilibrium. Let there be i=1,2,…,K parallel chains, each initialized and burned-in independently for $B_{i}$ updating steps before more samples are drawn at intervals. As K→∞ and all$B_{i}\to \infty$_,_ it can be shown that the ensemble is ergodic (tending in the limit) to $p\left(X\right)$[19].

An appealing advantage of running multiple chains is that these processes can be conducted concurrently with minimal coordination among tasks, as in the case of parallel Monte Carlo simulation. However, unlike parallel Monte Carlo simulation, a major concern with running multiple MCMC is that the overall reduction in runtime from parallelism can be limited by the portion of each chain to be discarded in the beginning of MCMC sampling for convergence purposes (i.e., burn-in). If every chain has to spend a significant proportion of its time in burn-in, this would place serious limitations on the performance of the algorithm, because it would not scale well with an increasing number of processors [4]. According to Amdahl’s (1967) law [20], if some portion *ρ* of steps, for 0<ρ≤1_,_ must be removed as burn-in from each chain, then the maximum speedup in computing through parallelization is (assuming that each step takes an equal amount of time):

(10) $$S\left(\rho \right)=\frac{\rho n+n}{\rho n+nK^{-1}}\underline{\underline{K\to \infty }}\;\;1+\frac{1}{\rho }$$

where *n* is the number of iterations after burn-in. Thus, parallel MCMC computing by virtue of running multiple chains is rewarding only when *ρ* is small. However, if *ρ* is large, the gain in computation through running multiple chains instead of a single long chain can be very disappointing.

Although running multiple Markov chains is theoretically straightforward, chains are not necessarily ergodic. Hence, some variant multiple MCMC methods have been proposed. For example, samples from multiple Markov chains may be confined to isolated modes if the target distribution is multi-modal, or the chains may mix poorly when there are strong correlations between variables. Unfortunately, the latter is a common problem of Gibbs samplers [21]. Hence, pooling samples from multiple short chains may not necessarily give a better representation of *p(X)* than using a single long chain. If several chains are drifting to disparate modes, they will tend to be strongly influenced by the chains that they confine, because the weights will not necessarily be proportional to their relative masses.

Several strategies have been proposed for handling the aforementioned issues for single chains, such as adaptive MCMC algorithms [16,22] and tempering [23,24]. Metropolis-coupled MCMC is an algorithm that is related to simulated tempering and tempered transitions [23,24]. It proceeds by simultaneously running a number of different Markov chains that are governed by different (but related) Markov chain transition probabilities. Occasionally, the algorithm “swaps” values between two different chains, with probability governed by the Metropolis algorithm to preserve the stationarity of the target distribution. These swaps can speed up convergence of the algorithm substantially [4]. Craiu et al. [25] targeted the posterior with an ensemble of chains, using the covariance of samples across all chains to adapt the proposal covariance for a set of Metropolis-Hastings chains. While these multiple-chain methods use synchronous exchange of samples to expedite convergence, Murray [26] proposed mixing in an additional independent proposal, representing some hitherto best estimate or summary of the posterior, and cooperatively adapting across chains. The idea is to construct a global best estimate of the posterior at any given step and then mix this estimate as a remote component with whatever local proposal that a chain has adopted. This does not preclude adaptive treatment or tempering of that local proposal. It also permits a heterogeneous blend of remote proposals, so that the ensemble of chains can mix well.

#### Parallelization within a single chain

By running multiple Markov chains, we often observe that samplers mix poorly and each chain may require a very long burn-in time. Hence, it would be preferable to develop parallelism within a single chain, instead of running multiple chains. As mentioned in the previous section, Markov chain simulation is an iterative procedure, in the sense that simulation of the next value of the chain depends on the current value. This creates difficulty for delivering parallelism for a single Markov chain. Nevertheless, we will show that a single chain can be parallelized, subject to assumptions of conditional independence in the model. The key is to identify such steps that can be implemented in parallel.

Consider a Bayesian model with *p* scale parameters $\sigma =\left(\sigma_{1},\sigma_{2},\sigma_{p}\right)$, where *p* can be equal to 1 in some cases, and *q* location parameters$\theta =\left(\theta_{1},\theta_{2},\theta_{q}\right)$. In MCMC sampling, each element is updated once per iteration using a kernel density that preserves the desired target posterior distribution$p\left(\sigma ,\theta |y\right)$Assume that updating *σ* is very fast (given some sufficient statistics regarding the current state of **θ**) but updating **θ** is highly time-consuming. This is typical of a multivariate normal distribution with a common scale parameter (or different groups of scale parameters) and a large number (say a few hundreds or thousands) of location parameters. In these cases, it would be preferable to parallelize the update steps for **θ** in order to gain speed up in computing. In theory, parallelization of the update of **θ** depends crucially on the conditional independence structure of the model. First, assume the simplest possible case, where $\theta_{i}⊥\theta_{j}|\sigma ,y,i\neq j$, meaning that the update of any particular $\theta_{i}$ will not depend on the state of any other $\theta_{j}$$\left(j\neq i\right)\text{.}$ Thus, all θ's can be updated in parallel by delivering subsets, say $q_{k}\text{,}$ of the elements in ***θ*** to the *K* CPU cores. For illustrative purpose, let there be only one *σ* but manyθ's. Then, after all parameters are given initial values, the parallel MCMC algorithm proceeds by repeatedly conducting the following steps:

➔ Master program:

(a) samples a new *σ*, given realization of **θ** and the data ***y***, and

(b) distributes the new *σ* to each process.

➔ Each process (*k*):

(a) updates a subset of *θ*s that have been assigned to it, conditional on *σ* and **y**,

(b) computes summary statistics for the updated θs, and

(c) passes the summary statistics back to the master program.

Often, the above algorithm works quite well when the *θ* are all independent of one another, given *σ* and *y*. In practice, however, such independence may not necessarily hold and strategies must be developed to deliver efficient parallel MCMC algorithms given specific dependence between elements [3].

### Applications

#### Parallel simulation for a single-parameter normal model

Consider a normal distribution model with unknown *μ* and known$\sigma^{2}\text{.}$ For a vector y of *n iid* observations, the likelihood is:

(11) $$p\left(\text{y}|\mu \right)=\overset{n}{\underset{i=1}{∐}}\left(\frac{1}{\sqrt{2\pi \sigma^{2}}}\exp\left(-\frac{\left(\text{y}-1\mu \right)'\left(\text{y}-1\mu \right)}{2\sigma^{2}}\right)\right)\text{.}$$

If a normal prior is assumed, that is,

(12) $$p\left(\mu \right)\propto \exp\left(-\frac{1}{2\tau_{0}^{2}}{\left(\mu -\mu_{0}\right)}^{2}\right)$$

where $\mu_{0}$ and $\tau_{0}^{2}$ are hyperparameters, it can be shown that the posterior density of *μ* is also normal [15]:

(13) $$p\left(\mu |y\right)=N\left(\frac{\frac{1}{\tau_{0}^{2}}\mu_{0}+\frac{n}{\sigma^{2}}\bar{y}}{\frac{1}{\tau_{0}^{2}}+\frac{n}{\sigma^{2}}},{\left(\frac{1}{\tau_{0}^{2}}+\frac{n}{\sigma^{2}}\right)}^{-1}\right)\text{.}$$

Intuitively, the posterior mean of *θ* is expressed as a weighted average of the prior mean ($\mu_{0}$) and of the sample mean ($\bar{y}$), with weights equal to the corresponding precisions, $\frac{1}{\tau_{0}^{2}}$ and $\frac{n}{\sigma^{2}}$, respectively. Because this is a single-parameter model, posterior samples of *μ* can be simulated in parallel by following the same algorithm as for Monte Carlo simulation.

**Figure 1:**
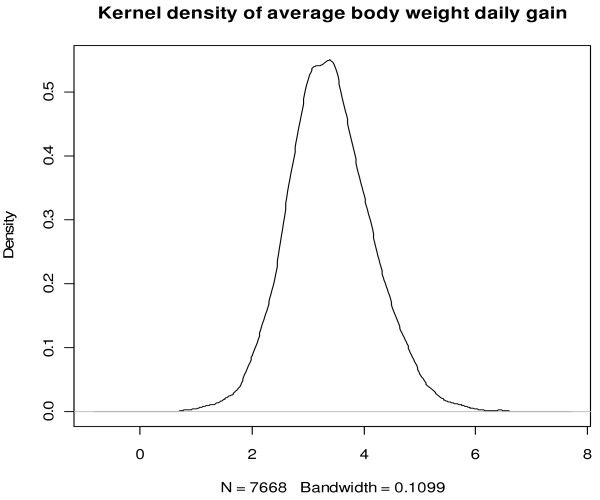
**Kernel density of average body weight daily gain measured in 7668 Angus cattle** .

The example data are average body weight daily gains (ADG) measured on 7670 Angus cattle. The kernel density of ADG is shown in Figure 1, which approximately suggests a normal distribution. Assume that we know, from previous studies, that the population variance of ADG is 0.58. In this example, the prior distribution is assumed to be normal with mean equal to 4.0 and variance equal to 1.0 (these are just guesses of the parameter values in the distribution of ADG). A parallel C program was used in this analysis (Appendix). To compile the parallel program, say using MPICH2, type: mpicc singNormMod_Parallel.c –o singNormP –lm [enter]. To conduct computing in parallel, type: mpirun –np xx ./singNormP [enter], where xx is the number of processors involved (or CPU cores). To estimate µ, we simulated a total of 1 000 000 values for μ, which were handled by K=10 processes, each generating 100 000 values and computing the partial sum. Then, the *K* partial sums were transferred back to process 0, where the Monte Carlo estimate was computed. The illustrative program only outputs the posterior mean and the standard deviation. The original program used in the demonstration also outputs minimum and maximum values, and quartiles. This part of the code is omitted in the Appendix for simplicity of demonstration. The computing was conducted on a DELL Precision workstation equipped with Intel® Xeon® CPU (3.20GHz), 16G memory, and cache size 6144KB.

The posterior mean was estimated to be 3.394, which corresponded very well to the sample mean of ADG of the 7670 Angus cattle (Table 1) because the impact of the prior on the posterior could be ignored given the very large sample size. The median and mean agreed well with each other and the first and third quartiles were also very similar (Table 1). These are indications that the posterior distribution of the mean of ADG is symmetric.

**Table 1:** Posterior summary statistics of average body weight daily gain based on a single-parameter normal model

**Sample set**	**Min**	**Q1**	**Median**	**Mean**	**Q3**	**Max**
1	3.357	3.388	3.394	3.394	3.400	3.431
2	3.356	3.388	3.394	3.394	3.400	3.429
3	3.352	3.388	3.394	3.394	3.400	3.43
4	3.357	3.388	3.394	3.394	3.400	3.436
5	3.353	3.388	3.394	3.394	3.400	3.432
6	3.355	3.388	3.394	3.394	3.400	3.43
7	3.355	3.388	3.394	3.394	3.400	3.431
8	3.354	3.388	3.394	3.394	3.400	3.428
9	3.356	3.388	3.394	3.394	3.400	3.431
10	3.358	3.388	3.394	3.394	3.400	3.433
**Pooled**	**3.352**	**3.388**	**3.394**	**3.394**	**3.400**	**3.436**

Min = minimum value; Q1 = first quartile; Q3 = third quartile; max = maximum value.

The above results were obtained from parallel computing on ten CPU cores.

The purpose of this example was to show parallel computing using the MPI (Message Passing Interface) library. The change in computing time for this example was, however, almost insignificant because sampling from a normal distribution is very quick. In addition, with parallel simulation, inter-process communication requires some extra time as overhead, which offset gains from parallel computing.

MPI is a language-independent communication protocol used to program parallel computers that is extensively used for high-performance computing. More specifically, MPI is a library of routines for creating parallel programs e.g., in C or Fortran 77, that allow users to create programs that can run on most parallel computer architectures. (Note that there is a language extension to Fortran90 called High Performance Fortran – HPF, which supports high-performance computing.) In the example code, the MPI library was used to handle inter-process communications in the C program. With MPI, each task can have its own local memory during computation (but multiple tasks can reside in the same physical machine and/or an arbitrary number of machines). Typically, tasks exchange data by sending and receiving messages but data transfer usually requires cooperation among processors, that is, a “send” operation must have a matching “receive” operation.

A few details about this program in the Appendix are described in the following. MPI_Comm_rand() is used to find out the ID of all participating processors and MPI_Comm_size() is used to get the number of participating processors. A common pattern of interaction among parallel processors is to use MPI_Send() and MPI_Receive() to allocate work among them. In the present example, however, this was done in a slightly different manner. MPI_Bcast() is used to send common parameter values (e.g., number of simulation steps) to all participating processors. Then, after each processor has finished its work, the subroutine MPI_Reduction() is used to sum up the posterior values from all processors. Subroutine MPI_Reduction() collects data from all processors, reduces the data to a single value (e.g., by summation), and then stores the results on the master process (and on all processes as well). There are several predefined operations that MPI_Reduction() can provide. In addition to summation, it can also conduct multiplication, and find minimum or maximum values. Finally, the master processor computes the means and standard deviation (and other posterior statistics, when relevant) for the mean of the normal model. Note that, in this illustration, we used sequential functions to generate random numbers (http://apps.nrbook.com/c/index.html), with process ID used as the random number seed. Preferably, one can use a parallel random number generator, such as the Scalable Parallel Random Number Generators (SPRNG) Library (http://sprng.cs.fsu.edu/).

### Running multiple chains for Bayesian LASSO modeling

In this example, we show how to parallelize multiple chains for the Bayesian LASSO (Least Absolute Shrinkage and Selection Operator) regression model [12,27]. In the whole-genome prediction context, consider the following linear model:

(14) y=1μ+Xβ+e,

where **y** is a vector of phenotypes, *μ* is an effect common to all observations, **β** is a vector of unknown marker effects, **X** is an incidence matrix, and ***e*** is a vector of residuals. The prior specification follows de los Campos et al. [12] but without the terms for additive genetic effects. An R package, BLR, was used to implement the Bayesian LASSO model [28].

The dataset used here consisted of 147 Angus cattle, each genotyped for 37 892 polymorphic SNP (Single Nucleotide Polymorphisms) markers and with estimated breeding values (EBV) for marbling score as response variable. In addition to running a single long chain of 100 000 iterations (after a burn-in of 1000 iterations), we also ran 10 chains, each consisting of 10 000 iterations (after a burn-in of 1000 iterations). All jobs were submitted and run on a Condor cluster at the University of Wisconsin – Madison [29]. This cluster provides 1860 cores for distributed parallel computing. Among them, 1847 run a Linux operation system and the remaining a Windows operation system. Memory size ranges from 256M to 214G: 7.04% (<1G), 67.58% (1-3G), 22.69% (4-8G), and 2.67% (>10G). A Perl script was used, that installs the R system and required libraries (such as the SuppDists package) onto remote nodes prior to the computing and then executes the Bayesian LASSO program. This Perl script served as the executable in the Condor job batch file.

**Figure 2:**
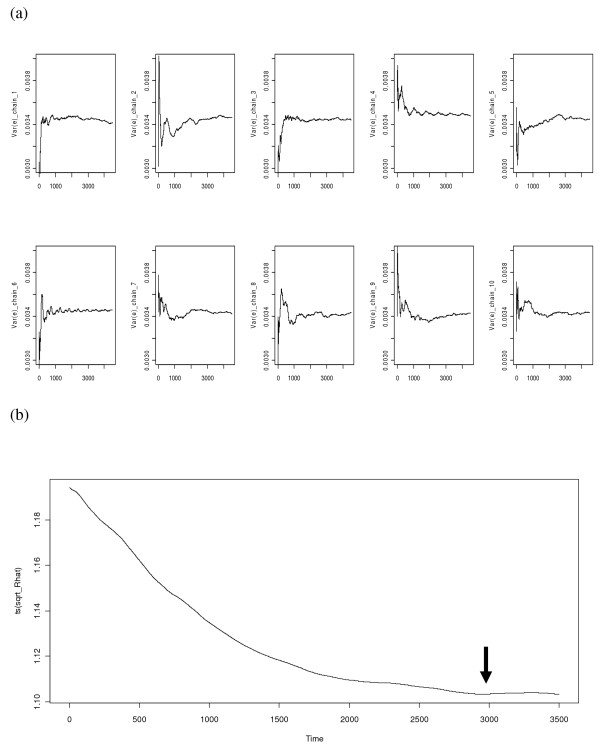
**Markov chain Monte Carlo convergence diagnosis.** (**a**) Trace plots of posterior samples of residual variance obtained within 4500 iterations from each of the 10 chains; (**b**) trace plot of shrink factor $\sqrt{\overset{⌢}{R}}$ according to the Gelman-Rubin method y.

For the multiple chains approach, each started with over-dispersed initial values (and with different seeds for the random number generator), and the chains converged after a certain number of iterations. Markov chain Monte Carlo convergence was examined using posterior samples of the residual variance collected from the first 4500 iterations of each chain. Trace plots of posterior samples of the residual variance showed that most chains tended to stabilize after 1000 iterations, and all approached 0.0034 (Figure 2a), which corresponds well to estimated residual variance in this example. The trace plot of the shrink factor $\sqrt{R^{2}}$ from the Gelman and Rubin method [17] suggested that a burn-in of 3000 iterations would be more appropriate, because $\sqrt{R^{2}}$ approached 1.00 after the first 3000 iterations (Figure 2b). With $\sqrt{R^{2}}\to 1\text{,}$ within-sequence variance dominates between-sequence variance, and all sequences escape the influence of starting points and traverse all target distributions. The same convergence diagnosis can be done for all model parameters. 

**Figure 3:**
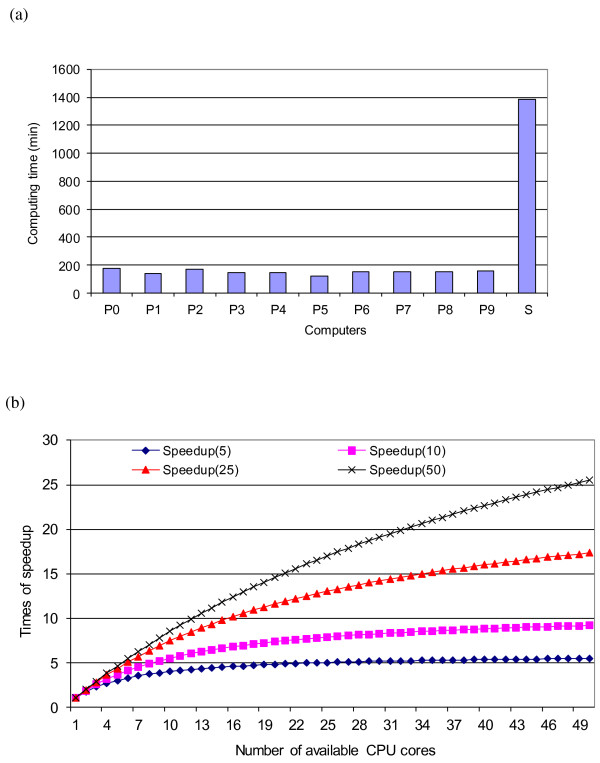
**Parallel vs. sequential computing of a Bayesian LASSO model for genome-enabled prediction of genetic merit.** (**a**) Comparison of computing time; (**b**) expected speedup by parallel computing with the chain length equal to ten times the burn-in length for the Markov chain.

The parallel computing took between 141 and 178 min to complete each of the 10 processes. The differences were due to varying CPU speeds and workloads on these computer nodes. In contrast, running a single chain with 100 000 iterations (after a burn-in of 1000 iterations) on a Linux workstation with similar specifications took 1386 min (Figure 3a). Thus, the reduction in runtime from parallel computing was approximately 7.78 fold. Posterior estimates of the model parameters were similar between the two computing approaches (data not presented).

When running multiple chains, the reduction in runtime is limited by the time required for burn-in. Let *b* denote the number of burn-in iterations that is required, and *n* be the number of iterations after the burn-in. Then, in a serial implementation, the chain will consist of *b* + *n* iterations in total. Let there be *K* processes running chains in parallel, each taking on an equal length of Markov chain (i.e., *b* + *n/K* iterations). Assuming each iteration takes the same amount of processing time, the reduction in runtime is given by:

(15) $$S\left(K\right)=\frac{b+n}{b+nK^{-1}}\underline{\underline{K\to \infty }}\;1+\frac{1}{b}\text{.}$$

The above is an alternative form of (10) with b=ρn and *K* treated as unknown. Ideally, if b=0, this means “perfectly parallel computing”, in which the potential reduction in runtime is *K-*fold. However, when *b* is a significant proportion of *n*, the actual speedup falls well short of its potential.

Practically, burn-in time is related to the mixing rate of the chain, which is related to the total length of the Markov chain. Let *n* = 10*b*, which is a useful rule-of-thumb in most practical situations. Then, equation (15) depends only on parameter *K*. Hence, we have $S\left(K=8\right)=\frac{11}{1+10/8}\approx 5$ and $S\left(K=16\right)=\frac{11}{1+10/16}<7$ respectively, for *K* = 8 and *K* = 16. As K→∞, the speedup is upper-bounded at:

(16) $$S\left(K\right)=\frac{b+10b}{b+10b\times K^{-1}}\overset{K\to \infty }{=}11.$$

Thus, when running multiple chains, each with a significant length of burn-in, the speedup does not scale well with the number of available CPU cores. Let n=t×b. Then, the speedup *S* is a function of *t* and *K*, as depicted in Figure 3b. Clearly, given the fixed relationship between *n* and *b*, the speedup will reach a plateau after the number of CPU cores reaches a certain level.

### Parallel BayesCpC for whole-genome prediction

**Figure 4:**
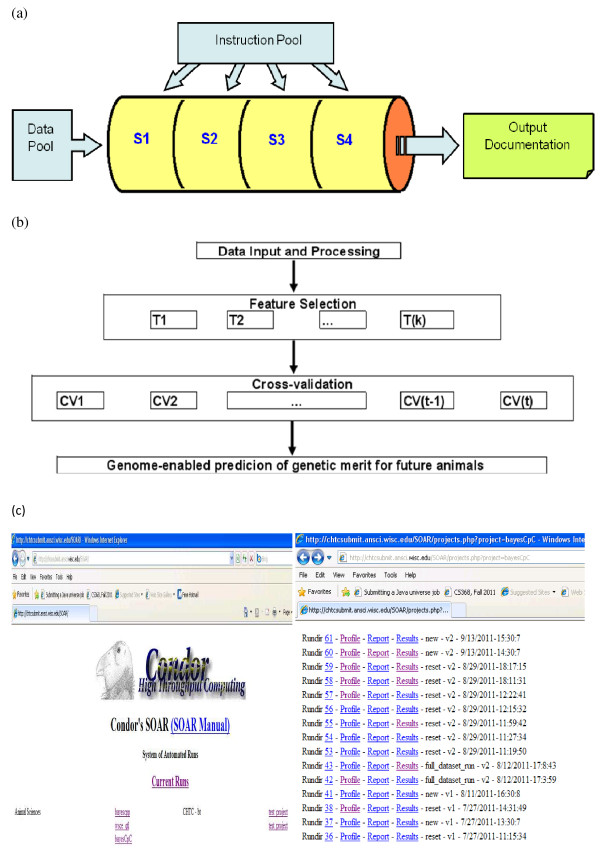
**Whole genome-enabled selection and evaluation (WGSE) pipelines and parallel BayesCpC.** (**a**) Graphic illustration of the WGSE pipelining; (**b**) workflow; (**c**) Condor SOAR webpage of the parallel BayesCpC pipeline.

Finally, we show a real application of parallel computing on genome-enabled prediction in beef cattle. The computing was implemented with a high-throughput computing pipeline called parallel-BayesCpC [30]. This is a high-throughput computing package and a member of the WGSE (Whole-Genome-enabled Selection and Evaluation) family [31] of distributed high-throughput computing pipelines. In computing, a pipeline is a set of data processing elements connected in series (i.e., the output of one element is the input of the next one) and the elements of a pipeline can be executed in parallel or sequentially. Typically, pipelining increases the computing throughput. In the context of whole-genome prediction, the pipeline that we have developed can automate all steps involved in the computing and decision making for whole-genome prediction, which includes and is not limited to data input and quality control, model feature selection (FS) if applicable, post-FS statistical inference and cross-validation (CV), and output and documentations (Figure 4a). The parallel-BayesCpC package is so named because it uses the BayesCπ model for FS and the BayesC $\left(\pi =0\right)$ model for post-FS statistical inference and CV (Figure 4b). This package can reside on both Condor and OSG (Open Science Grid) and is provided with a Condor SOAR web interface for automatic scheduling of jobs and storage of output files (Figure 4c). In Condor, for example, job dependency can be conveniently handled as so-called “DAGMan jobs” (Appendix – b). Note that the user does not need to know how to write Condor job batch files as all these files will be automatically produced by scripts of BayesCpC based on the user’s input. 

For simplicity of illustration, consider a linear model with only the overall mean and marker effects:

(17) $$y_{i}=\mu +\overset{p}{\underset{j=1}{\Sigma }}z_{\mathrm{ij}}\alpha_{j}+e_{i}$$

where $y_{i}$ is the phenotype (or estimated genetic value) of the *i*^th^ animal; *µ* is the overall mean; *a*_*j*_ is the substitution effect associated with the *j*^th^ SNP (j=1,…,p); $z_{\mathrm{ij}}$ is a variable corresponding to the genotype of the *j*^th^ SNP (0, 1, 2) for the *i*^th^ individual, and $e_{i}∼N\left(0,\sigma_{e}^{2}\right)$ is a residual term, where $\sigma_{e}^{2}$ is the residual variance. *A priori*, the BayesCπ model assumes that the effect of an SNP is null with probability π, or that it follows a normal distribution, $N\left(0,\sigma_{\alpha }^{2}\right)\text{,}$ with probability 1-π. That is,

(18) $$\alpha_{i}|\pi ,\sigma_{\alpha }^{2}\{\begin{array}{l} ∼N\left(0,\sigma_{\alpha }^{2}\right)\;\text{with probability}\;\left(1-\pi \right)\\ =\;\text{0 with probability}\;\pi \; \end{array}\text{.}$$

Here, $\sigma_{\alpha }^{2}$ is a variance common to all non-zero SNP effects, which is assigned a scaled inverse chi-square distribution, $\chi^{-2}\left(v_{\alpha },{\text{s}}_{\alpha }^{2}\right)\text{.}$ Furthermore, the value of *π* is unknown and needs to be inferred, given the prior distribution of *π* that is taken as uniform between 0 and 1, $\pi ∼Uniform\left(0,1\right)\text{.}$ A Bernoulli indicator variable, $\delta_{j}\text{,}$ is introduced to facilitate sampling from the mixtures of the SNP effects, $p\delta_{i}|\pi =\pi^{1-\delta_{i}}{\left(1-\pi \right)}^{\delta_{i}}\text{.}$ Hence, unconditionally, the variable $\alpha_{j}$ follows a multivariate-t distribution, $t\left(0,S_{\alpha }^{2},\upsilon_{\alpha }\right)\text{,}$ if $\delta_{j}=1\text{,}$ or equals zero otherwise [32]. Posterior inference of unknown parameters in the Bayesian model via Markov chain Monte Carlo implementation is described by Habier et al. [11].

With a subset of, say k≤p markers selected in a certain iteration of the MCMC for the BayesC *π* model, then the next iteration assumes that all the *k* selected SNP have non-null effects on the quantitative trait. The above defines BayesC model with π=0, which takes the same form as (15) but with π=0 and *p* replaced by *k*. Posterior inference in BayesC $\left(\pi =0\right)$ is the same as for BayesCπ, except that *π* is fixed at zero and sampling indicator variables is no longer relevant.

Typically, *K*-fold CV is often used to evaluate predictive models, in which the whole dataset is divided into *K* portions of approximately equal size. The model is then trained in the set of *K-1* portions of the data and predicted in the remaining one portion. Portioning of training and testing sets is then rotated *K* times in each CV experiment. Furthermore, each CV experiment can be randomly replicated a number of times in order to increase the stability of model evaluation (but we did not do that in the present study).

As an example, we used the parallel BayesCpC package to select the optimal number of SNP to predict rib eye area in a beef cattle population. The data consisted of 2919 animals each with estimated breeding values for rib eye area and genotypes obtained from the Illumina 50K SNP Beadchip. After data editing and data quality control, 46 723 polymorphic SNP markers were used. The optimal panel search started with the top 50 SNP markers according to the posterior model probability for having a non-zero effect for this marker, and then increased from the top 100 to the top 3000 markers at an increment of 100 markers each time. We did not exhaust all possible panels beyond 3K because the prediction accuracy showed a constant trend of decrease after the panel reached 1000 SNP.

In the parallel-BayesCpC package, MPI is used for data quality control and parallel MCMC is employed by both FS and CV. In our example, distributed jobs were submitted to a local Condor pool with 128 cores. These are Linux workstations, with Intel® Xeon® CPU (2.67 GHz), > 8G memory per slot and a cache size of 12 288 KB. The submit machine is also a Linux workstation with Intel® Xeon® CPU (3.00 GHz), 16G memory and 6144 KB cache size. Each parallel MCMC chain for feature selection consisted of 10 000 iterations after a burn-in of 2000 iterations, thinned every one-tenth. Each parallel MCMC for CV consisted of 25 000 iterations, after a burn-in of 5000 iterations, thinned every one-tenth.

We examined three computing strategies to search for an optimal panel size for predicting genetic merit using SNP markers. The first strategy executed 30 meta-jobs in parallel, each consisting of one round of parallel FS jobs using all SNP markers and one round of parallel post-FS inference and CV for a specific panel (X = 50, 100, 200, …, 3000, respectively). In the second strategy, one round of parallel FS jobs was executed, followed by 30 rounds of parallel post-FS inference and CV jobs conducted sequentially for all panel sizes. Parallel CV jobs for the 500-SNP panel started only when parallel CV jobs for the 400-SNP jobs had finished. The third strategy consisted of 30 meta-jobs executed in series, with each meta-job consisting of one round of parallel FS jobs using all SNP markers and one round of parallel post-FS inference and CV for a specific panel. The difference between the second and the third strategy is that different optimal panel sizes were selected based on the same set of FS results with the second computing strategy but each panel was selected based on a different set of FS results with the third computing strategy.

**Figure 5:**
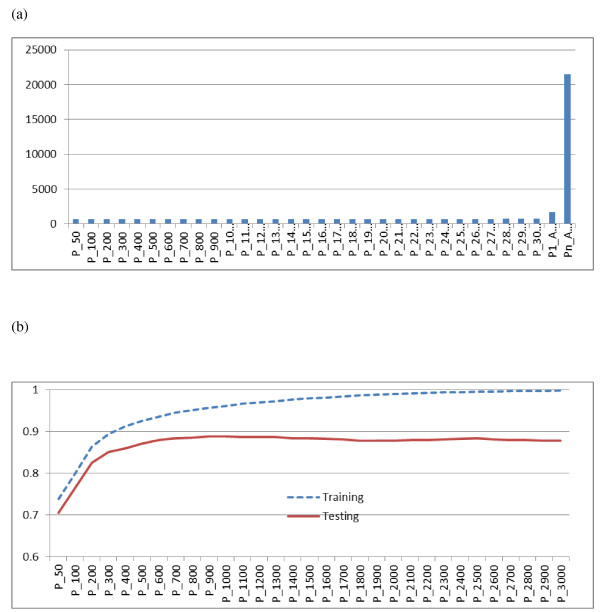
**Computing time by three parallel computing strategies (a) and prediction correlations by the first strategy (b).** Three parallel computing strategies were used in search of optimal SNP panel sizes for predicting genetic merit. The first strategy executed 30 meta-jobs in parallel, each consisting of one round of parallel feature selection (FS) jobs using all SNP markers and one round of parallel post-FS inference and CV for a specific panel (X = 50, 100, 200, …, 3000, respectively); In the second strategy, one round of parallel FS jobs was executed, followed by 30 rounds of parallel post-FS inference and CV jobs conducted sequentially for all panel sizes (P1_A); The third strategy consisted of 30 meta-jobs executed in series, with each meta-job consisting of one round of parallel FS jobs using all SNP markers and one round of parallel post-FS inference and CV for a specific panel (Pn_A).

There were very significant differences in computing time between the three computing strategies (Figure 5a). The first strategy consumed the least time (mostly completed within 12 h), because it used more features of parallel computing, but it also required far more slots for computing (in total 90 slots were needed). The second strategy avoided the use of many slots because it ran only one round of parallel FS jobs and then executed 30 rounds of parallel post-FS inference and CV jobs sequentially based on the same set of FS results. We ran three parallel chains for FS and also used 3-fold CV to evaluate prediction accuracy and, hence, only three slots were needed for this strategy. Because CV jobs on a subset of markers typically ran much faster than a FS job on all markers, it was computationally efficient to run these CV jobs sequentially. The computing time necessary for the second strategy was approximately two times greater than for the first strategy. However, the third strategy consumed the greatest amount of time (over two weeks). With this strategy, jobs for different panel sizes were executed in series but FS jobs and post-FS inference and CV jobs for each panel size were executed in parallel. If all these jobs were executed sequentially, the computing time necessary would exceed one month and half, and this is definitely not optimal. Comparatively, the first computing strategy was twice as fast as the second strategy and 29 times faster than the third strategy.

**Figure 6:**
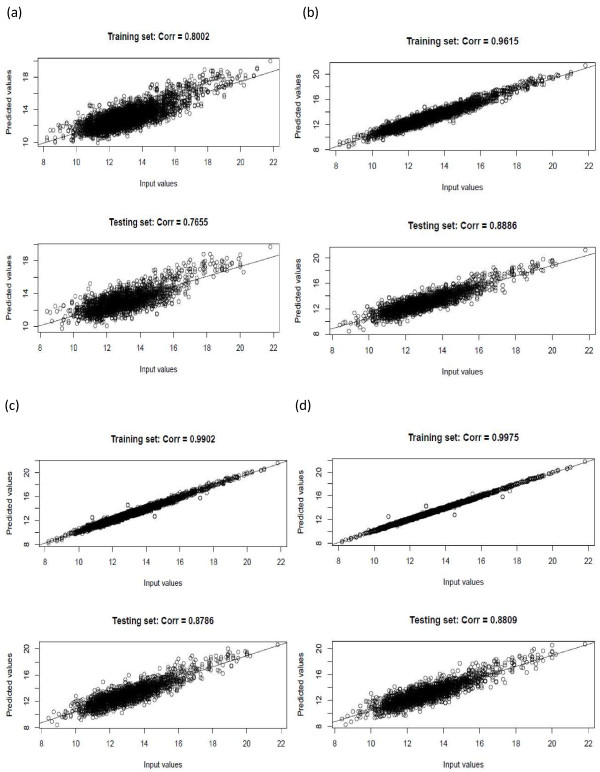
**Prediction accuracies obtained from different panel sizes.** (**a**) 100 SNP; (**b**) 1000 SNP; (**c**) 2000 SNP; (**d**) 3000 SNP.

Despite the differences in computing times, predictions obtained with the three computing scenarios were highly comparable. The correlation between estimated breeding values for rib eye area and their fitted values in the training set (referred to as fitting accuracy hereafter) increased almost monotonically with panel size, until it plateaued with a panel size of around 2000 SNP (Figure 5b). However, the correlation between the estimated breeding values and their predicted values in the testing set (referred to as predictive accuracy hereafter) reached its peak (0.8886) with a panel size of 1000 SNP, and then went down slightly. The highest predictive accuracy was observed with 500 to 1500 selected markers. The decrease in predictive accuracy with > 1000 SNP possibly reflected over-fitting, which, in this case, occurred much before the panel size exceeded the training population size (i.e., around 2000 animals). Hence, with Bayesian regression models, prediction using more SNP may not necessarily give better results than prediction using a smaller panel. A model that describes the training set well does not necessarily yield the best predictions when generalized to the population. This is referred to as poor generalization in machine learning [33]. The fitting and prediction accuracies are illustrated in Figure 6 for various panel sizes. 

**Figure 7:**
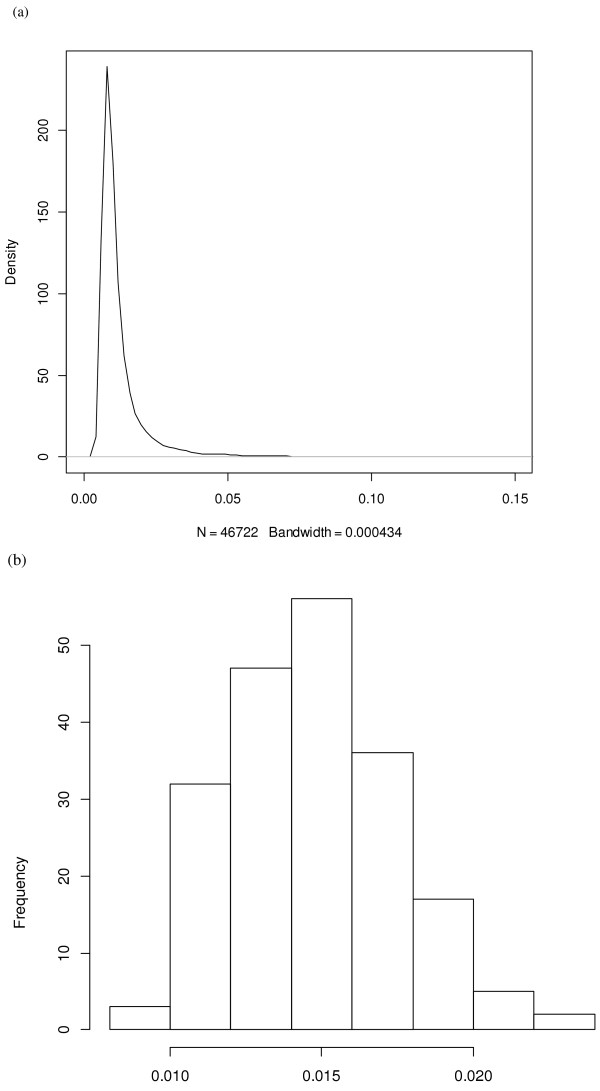
**Histogram of posterior estimates of 1-π in the BayesCπ model for feature selection.** The results were obtained from feature selection using computing strategy II, that is, one round of parallel FS jobs was then executed, followed by 30 rounds of parallel post-FS inference and CV jobs conducted sequentially for all panel sizes.

In the Bayes CpC procedure, the BayesC *π* model postulates that a portion *π* of all SNP have zero effect. In a high-density SNP panel, *π* is typically expected to be large, meaning that the portion of “signal” SNP, 1-π, is small (Figure 7), so the chance of over-fitting is diminished. Using the Illumina Bovine50K SNP genotypes, the posterior mean (standard deviation) of 1−π was 0.0148 (0.0027). In prediction, the best predictions were obtained with 500 to 1500 selected SNP, supporting an optimal predictive ability with 1.07% to 3.21% selected markers. Interestingly, this optimal range covered the posterior mean of 1−π, which is the portion of markers estimated to have non-zero effects on the trait. This result differs somewhat from what we obtained using 3K SNP panels (data no published yet). For the 3K genotypes, the estimated number of SNP having non-zero effects based on BayesC *π* in the training set did not correspond to the number of SNP in the optimal SNP panel for prediction. Hence, we postulate that parameter *π* in a BayesC *π* model may not provide information on the size of an optimal SNP panel for prediction for small panels but could be informative for a higher density of markers.

With the Bayesian regression models explored here, feature selection may be important since a model with all SNP does not necessarily give the best predictions. This situation is unlike ridge regression best linear unbiased prediction [34] or prediction using the G-BLUP method [35], for which a model with all markers would typically be favored. While selecting models of varying dimensions may be an issue to explore, it brings tremendous challenges to computing, particularly when the dataset is large. In this regard, high-performance computing offers a markedly competitive edge, not only in reducing computing time but also in tuning optimal models for whole-genome prediction.

## Discussion

To date, almost all statistical software packages for animal breeding and genetics have been developed for serial computation. In such programs, only one instruction is executed at a time and after that instruction is finished, the next instruction begins. Hence, serial computing performance depends heavily on the speed (clock frequency) of CPU, and the runtime of a program is approximately equal to the number of instructions multiplied by the average time per instruction. Keeping everything else constant, higher clock frequency leads to faster computing speed and thus decreased runtime for all computation-bounded programs [36]. This was the situation with the performance enhancement of microprocessors based on a single CPU from the 1980s to the early 2000s. However, rate of improvement has slowed down since 2003 due to hardware limitations incurred by energy consumption and heat dissipation. On the other hand, parallel computing has gained impetus as a result of increasingly available multiple-core computers, computer clusters, and networking and has been referred to as the concurrency revolution [37]. In theory, multiple threads of execution can cooperate to complete the work faster than a serial setting.

Parallel computing uses multiple processing elements concurrently to solve a problem. To implement parallel computing, one first needs to break the problem into discrete “chunks” of work, so that they can be distributed to run on multiple processors. This is known as task decomposition or partitioning. The next fundamental step in designing the parallel algorithm involves identifying independencies that are assumed explicitly in the model. Without loss of generality, let *P*_*i*_ and *P*_*j*_ be two program jobs, where *i* < *j* indexes the order of execution. Bernstein’s conditions [38] can be used to identify whether or not the two jobs are independent and can be executed in parallel [39]. Let *I*_*i*_ and *O*_*i*_ be the input variables and output variables, respectively, of *P*_*i*_. Likewise, the same definitions hold for *I*_*j*_ and *O*_*j*_ of *P*_*j*_ . Then, *P*_*i*_ and *P*_*j*_ are independent if they satisfy (1) $I_{j}\cap O_{i}=\emptyset \text{,}$ (2) $I_{i}\cap O_{j}=\emptyset \text{,}$ and (3) $O_{i}\cap O_{j}=\emptyset \text{.}$ Violation of condition (1) introduces a flow dependency because the results from the first job (*P*_*i*_) are used by the second job (*P*_*j*_). Violation of condition (2) introduces an anti-dependency, because *P*_*j*_ would overwrite a variable needed by *P*_*i*_ . The third condition represents an output dependency: when two statements write to the same location, the final result is determined by the last executed statement.

MCMC algorithms have revolutionized the application of Bayesian inference, because it tackles a large range of complex inferential problems that were previously not considered possible, tractable. In the meantime, statisticians are becoming ever more ambitious in the range (complexity) of models they consider and MCMC algorithms for large complex models often require enormous amounts of computing power. Consequently, effective exploitation of parallel algorithms is of high relevance to Bayesian computation. A difficulty, however, is that MCMC algorithms are serial by nature and do not easily migrate onto a parallel system. Nevertheless, various strategies can be used to design parallel MCMC methods. The key is to identify steps with data independence or conditional independence on which parallelism can reside. Several algorithms or strategies exist for running parallel MCMC, which is straightforward and it requires minimal inter-process communication. However, poorly mixing MCMC algorithms with long burn-in periods are not ideally suited to this situation, because a long period of burn-in must be repeated on every available CPU core. Thus, it is often desirable to explore strategies for parallelism within single chains.

The actual performance of parallel MCMC depends on several issues. Among them, inter-process communication is a primary factor. Many parallel applications require processes to share data with each other, which is known as inter-task communication and implies overhead. Inter-task communication can offset the gain in computing speed from parallel computing, because it frequently requires some type of synchronization between tasks, causing processes to spend time “waiting”. In the worst case, competing communication traffic can saturate the available network bandwidth, leading to poor parallel computing performance. Thus, there is always a need to balance the distribution of work among tasks. There is no simple rule for this type of load balancing. Ideally, all tasks are to be kept busy all of the time, so that task idle time is minimized [9,10].

The ratio of computation to communication is qualitatively measured by the concept of granularity (https://computing.llnl.gov/tutorials/parallel_comp/). On one hand, a low computation to communication ratio (fine-grain parallelism) facilitates load balancing, as relatively small amounts of computational work are done between communication events but this can imply high communication overhead and less opportunity for performance enhancement, because communication and synchronization between tasks may take longer than the computation. On the other hand, a high computation to communication ratio (coarse-grain parallelism) allows more opportunity for performance enhancement; because relatively large amounts of computational work are done between communication and synchronization events. However, it is difficult to balance loads efficiently with coarse-grain parallelism and computing time may differ dramatically between computer cores. Therefore, there is a tradeoff between computing and communication and the optimal granularity depends on the problem at hand. In most parallel MCMC problems, it is advantageous to have coarse granularity because the overhead associated with communication and synchronization is high relative to execution speed, but fine-grain parallelism can sometimes help reduce overhead due to load imbalance.

## Conclusions

In this paper, we have shown the principles and examples of parallel MCMC, with applications to whole-genome prediction of breeding values. Parallel computing operates on the principle that a large problem can be split into smaller components and solved concurrently (i.e., “in parallel”), each on a separate processor (or CPU core). In the context of parallel MCMC, two basic algorithms exist: running multiple chains and parallelism within a single chain, yet some variants can be useful as well. In principle, all Bayesian models can be parallelized in computing but the associated algorithms and strategies may differ, leading to varied computing efficiencies. Although many technical details have yet to be explored, we expect that the use of parallel MCMC methods will revolutionize computational tools for research and breeding programs for animals in the post-genome era.

## Appendix

**(a)** Example C code using MPI for parallel simulation of a single-parameter normal model with unknown mean and known variance

/* This is an example C program using MPI for parallel computing of *

* a single-parameter normal model with unkown mean and known *

* variance. For illustration purpose, the step for data input is omitted. *

* Instead, the sample mean and standard deviation, as well as the prior *

* mean and standard are used. *

* Contact: X-L Wu, nick.wu@ansci.wisc.edu; UW-Madison, 09-12-2011 */

#include < stdio.h>

#include < math.h>

#include < mpi.h>

#include “ranNum.h”

main(int argc, char **argv)

{int proc_id, root_process, nprocs, ierr, niters, i;

double xi, xi2, sum, psum, sum_xi2, psum_xi2;

double tar0, tar1, sdn, tarn, varn, mun, mumu, sdmu;

MPI_Status status;

/* Prior mean and standard deviation */

double mu0 = 4.000;

double sd0 = 1.000;

/* sample size, mean and variance */

int nind = 7670;

double mu1 = 3.394;

double sd1 = 0.580;

/* compute posterior statistics */

tar0 = 1.0/(sd0*sd0);

tar1 = (1.0*nind)/(sd1*sd1);

varn = 1.0/(tar0 + tar1);

sdn = sqrt(varn);

mun = varn * (tar0*mu0 + tar1*mu1);

/* Parallel simulation begins, starting from here */

/* process 0 as the root process. */

root_process = 0;

/* Replicate this process to create parallel processes. */

ierr = MPI_Init(&argc, &argv);

/* Allocate memory for random seed variable*/

long* idum;

MPI_Alloc_mem(sizeof(long), MPI_INFO_NULL, &idum);

/* Find out process ID and number of participating processes. */

ierr = MPI_Comm_rank(MPI_COMM_WORLD, &proc_id);

ierr = MPI_Comm_size(MPI_COMM_WORLD, &nprocs);

/* Root process gets the number of simulation steps */

if(proc_id == root_process) {

printf(“Please enter the number of simulation steps: ”);

scanf(“%i”, &niters);}

/* Broadcast the number of simulation steps to all participating processes */

ierr = MPI_Bcast(&niters, 1, MPI_INT, root_process,MPI_COMM_WORLD);

/*process id as the random number seed */

*idum = proc_id;

/* Each process computes a partial sum of simulated values */

psum = 0;

psum_xi2 = 0;

for(i = proc_id + 1; i < niters + 1; i + = nprocs) {

xi = mun + sdn * randomnormal(idum);

xi2 = xi * xi;

psum = psum + xi;

psum_xi2 = psum_xi2 + xi2;}

printf(“proc %i computes: %f\n”, proc_id, (float)psum);

/* Do a reduction in which all partial sums are combined into the grand sum */

ierr = MPI_Reduce(&psum, &sum, 1, MPI_DOUBLE,

MPI_SUM, root_process, MPI_COMM_WORLD);

ierr = MPI_Reduce(&psum_xi2, &sum_xi2, 1, MPI_DOUBLE,

MPI_SUM, root_process, MPI_COMM_WORLD);

/* Finally, the root process prints posterior mean and standard error of mu. */

if(proc_id == root_process) {

mumu = sum / niters;

sdmu = sqrt((sum_xi2 - niters*mumu*mumu)/(niters-1));

printf(“The posterior mean and variance of mu is %f and %f\n”, (float)mumu,(float)sdmu);}

/* Close down this processes. */

ierr = MPI_Finalize();}

**(b)** Condor job batch files (Note: these Condor scripts were written automatically be a R scripts based on a input parameter file. Using the BayesCpC package, a user does not need to write this type of Condor job batch files.)

# Condor DAGMan job

JOB input job_InputData

JOB selection job_Selection

SCRIPT POST selection /usr/local/wgse_beta/V2/cmd_postscript_SelectionSummary

JOB validation job_Validation

SCRIPT POST validation /usr/local/wgse_beta/V2/cmd_postscript_OutputResults

PARENT input CHILD selection

PARENT selection CHILD validation

# Data input and quality control

Universe = vanilla

Executable = /usr/local/wgse_beta/V2/rungeneric.pl

Arguments = ‐‐new ‐‐type = R ‐‐tarball = built-sl5-R-2.10.1.tar.gz ‐‐installfrom = R-2.10.1 ‐‐cmdtorun = p1_data_input_n_processing.R ‐‐unique = input

Log = step_input.log

Output = step_input.out

Error = step_input.error

notification = NEVER

should_transfer_files = YES

when_to_transfer_output = ON_EXIT

transfer_input_files =(data path and files are omitted), /home/nickwu/BayesCpC/V2_UW/data/datafile.csv, /usr/local/wgse_beta/V2/rungeneric.pl, /usr/local/wgse_beta/V2/SLIBS.tar.gz, /usr/local/wgse_beta/V2/cmd_data_input_n_processing, /usr/local/wgse_beta/V2/p1_data_input_n_processing.R,

Queue

# Feature selection

Universe = vanilla

Executable = /usr/local/wgse_beta/V2/rungeneric.pl

Arguments = ‐‐new ‐‐type = R ‐‐tarball = built-sl5-R-2.10.1.tar.gz ‐‐installfrom = R-2.10.1 ‐‐cmdtorun = p4_BayesCpC_validation.R ‐‐unique = validation_1 1

Log = step_validation.$(process).log

Output = step_validation.$(process).out

Error = step_validation.$(process).error

notification = NEVER

should_transfer_files = YES

when_to_transfer_output = ON_EXIT

transfer_input_files = .linkPar, /usr/local/wgse_beta/V2/rungeneric.pl, /usr/local/wgse_beta/V2/SLIBS.tar.gz, /usr/local/wgse_beta/V2/cmd_cross_validation, /usr/local/wgse_beta/V2/p4_BayesCpC_validation.R,

Queue

Arguments = ‐‐new ‐‐type = R ‐‐tarball = built-sl5-R-2.10.1.tar.gz ‐‐installfrom = R-2.10.1 ‐‐cmdtorun = p4_BayesCpC_validation.R ‐‐unique = validation_2 2

Queue

Arguments = ‐‐new ‐‐type = R ‐‐tarball = built-sl5-R-2.10.1.tar.gz ‐‐installfrom = R-2.10.1 ‐‐cmdtorun = p4_BayesCpC_validation.R ‐‐unique = validation_3 3

Queue

# Post-FS inference and cross-validation

Universe = vanilla

Executable = /usr/local/wgse_beta/V2/rungeneric.pl

Arguments = ‐‐new ‐‐type = R ‐‐tarball = built-sl5-R-2.10.1.tar.gz ‐‐installfrom = R-2.10.1 ‐‐cmdtorun = p4_BayesCpC_validation.R ‐‐unique = validation_1 1

Log = step_validation.$(process).log

Output = step_validation.$(process).out

Error = step_validation.$(process).error

notification = NEVER

should_transfer_files = YES

when_to_transfer_output = ON_EXIT

transfer_input_files = .linkPar, /usr/local/wgse_beta/V2/rungeneric.pl, /usr/local/wgse_beta/V2/SLIBS.tar.gz, /usr/local/wgse_beta/V2/cmd_cross_validation, /usr/local/wgse_beta/V2/p4_BayesCpC_validation.R,

Queue

Arguments = ‐‐new ‐‐type = R ‐‐tarball = built-sl5-R-2.10.1.tar.gz ‐‐installfrom = R-2.10.1 ‐‐cmdtorun = p4_BayesCpC_validation.R ‐‐unique = validation_2 2

Queue

Arguments = ‐‐new ‐‐type = R ‐‐tarball = built-sl5-R-2.10.1.tar.gz ‐‐installfrom = R-2.10.1 ‐‐cmdtorun = p4_BayesCpC_validation.R ‐‐unique = validation_3 3

Queue

## Competing interests

The authors declare that they have no competing interests.

## Authors' contributions

XLW worked out the conception and design of this study, developed the pipeline, analyzed the results and wrote the article. CS and TMB helped with programming, data analysis and proof-read the article. KAW, GJMR, NDLG, and DG were involved in the design of this study, discussion of the results, and proof-reading of this article. All authors read and approved the final manuscript.
